# MatricS—A novel tool for monitoring professional role development in surgical disciplines

**DOI:** 10.3389/fsurg.2022.1009391

**Published:** 2022-10-14

**Authors:** U. Necknig, H. Leyh, R. Waidelich, L. Gernhold, J. Kiesewetter, M. Weidenbusch

**Affiliations:** ^1^ Urologische Praxis Lindenberg, Lindenberg, Germany; ^2^ Deutsche Gesellschaft für Urologie, Junior Akademie, Düsseldorf, Germany; ^3^ Urologische Klinik und Poliklinik, LMU University Hospital, Munich, Germany; ^4^ Institute of Medical Education, University Hospital, LMU Munich, Munich, Germany

**Keywords:** medical education, mentoring, postgraduate, role development, CanMeds

## Abstract

**Introduction:**

Mentoring is an effective method for human resource development. Monitoring the process is important for individual mentee/mentor pairs as well as for program directors. Due to individual personality differences of both mentees and mentors and their respective interactions, it is challenging to monitor the individual development process of mentees in a structured manner. This study investigates to what extent a novel instrument, the mentee-based assessment tool for role development of interpersonal competencies in surgical professions (MatricS) can adequately monitor the professional role development process of residents during an established mentoring program.

**Material and methods:**

In a prospective longitudinal study, the competence development of 31 mentees in two subsequent cohorts was assessed by a modified role matrix based on Canadian Medical Education Directives for Specialists. The evaluation focused on three defined roles (D, developer; N, networker; M, multiplicator) at three levels (private, employer-related, national/international) with four stages of development. For validation of mentee self-assessments, the assessments of the respective mentors were recorded alongside. For correlation analyses, Pearson coefficients were calculated, pre-post-comparisons were done by paired t-tests; significance was assumed at *p* < 0.05, respectively.

**Results:**

Mentee self-assessments overall correlated well with the objective mentor assessments (Pearson's *r* 0.8, *p* < 0.001). Significant correlations of this magnitude were found for both individual cohorts as well as for all individual roles. The mentees acquired competencies in all roles indicated by significant increases of corresponding MatricS scores. The largest competency gains (mean ± SD) were found in the role D (start: 1.30 ± 0.77, end: 2.13 ± 0.83, *p* < 0.001). The majority of mentees achieved the prespecified target competency level in >75% of all roles and levels.

**Conclusion:**

The role development process during mentoring can be reliably monitored by using MatricS. MatricS scores highly correlate between mentees and mentors, indicating that mentee self-assessments are suitable and sufficient for monitoring. These findings help to lessen the work burden on senior surgeons and thus can help to increase the acceptance of mentoring programs in surgical disciplines.

## Introduction

It is currently forecast that one in three specialist positions across all disciplines will remain unfilled by 2030 (1). In order to counteract the impending shortage of specialists in the medical field, the education and training of physicians is once again coming into focus. The extensive technological and scientific changes in today's globalized world require residents to acquire wide-ranging competencies to properly perform the diverse professional activities of a surgeon (2, 3). The acquisition of these competencies goes beyond the previous level of cognitive-knowledge-oriented teaching of theoretical surgical training content and psychomotor-knowledge-oriented teaching of practical surgical skills (4).

As a result, many countries have adopted multidimensional, competency-based training programs to train their junior staff (5, 6). Some of these programs are based on the Canadian Medical Education Directives for Specialists (CanMEDs) model and define roles for which residents should be trained today (7). However, there is considerable uncertainty about how to teach the competencies for each role most effectively (3, 8, 9).

Mentoring has been known as an effective method of staff development since ancient times (10) and is becoming increasingly popular in medicine as an integral part of junior staff development as “training-near-by-the-job” (11). In urology, the Roadmap mentoring program was launched in 2005 (12) and has since supported physicians in their skill development (13, 14). The program aims to help mentees plan their careers at different skill levels and stages.

Because mentoring is resource intensive, quality control measures are needed to ensure appropriate use of time and money. While standardized instruments are available for quality assessment (15), most mentoring programs lack process evaluation (11).

However, monitoring the mentoring process is as important for individual mentee/mentor pairs as it is for mentoring program leaders (16). Process data can help identify mentees, that struggle to achieve the goals of the mentoring program during the ongoing program. This offers mentees and mentors the chance to tailor their mentoring relation to the individual needs of the mentee as theses needs become apparent during the program. Because of the individual personality differences of both mentees and mentors and their interactions, it is challenging to monitor the individual development process of each mentee in a structured way (17). Although some tools already exist for this purpose (18), their use is often mentor-based and/or resource-intensive, and these tools generally cannot meet the demands of senior surgeons and their schedules. An alternative to mentor-based assessment may be self-assessment by mentees. To our knowledge, there are no data on the adequacy and thus the applicability of mentee self-assessments to monitor the mentoring process, especially with emphasis on the professional role development of mentees. This study therefore investigates the extent to which a novel instrument, the Mentee-based Assessment Tool for Role Development of Interpersonal Competencies in Surgical Professions (MatricS), can adequately monitor the professional role development process of residents during an established mentoring program.

## Material and methods

In a prospective longitudinal study, the competence development of 36 mentees coached by 12 mentors in two subsequent program cohorts (2017/18, cohort 1; and 2019/20, cohort 2) was assessed by a modified role matrix based on CanMEDs. All mentees were residents in urology, mean age was 30.4 years, 20 mentees (56%) were women, 23 mentees (64%) were in the first half of their residency, eight mentees (22%) were employed at university hospitals. Nine mentors were chief of service in urology and the remaining three mentors were attending physicians in urology; 2 mentors worked in university hospitals, the remainder in academic teaching hospitals. The second cohort was partly organized online due to the Covid-19 pandemic. Informed written consent for participation in the mentoring program including the scientific analysis of the anonymous development matrix data was obtained from all participants prior to the start of the program. In short, the program “Urology Roadmap” runs for one and a half years, during which three meetings occur with all mentors and mentees together (entry, half-time, and exit meeting). Between these three conventions, mentee-mentor groups will meet individually, the timing and frequency of these meetings remains at the discretion of the corresponding groups. Typically, one group consists of three to four mentees with one mentor, so that the program features elements of peer-mentoring as well as classical mentoring.

**Figure 1:**
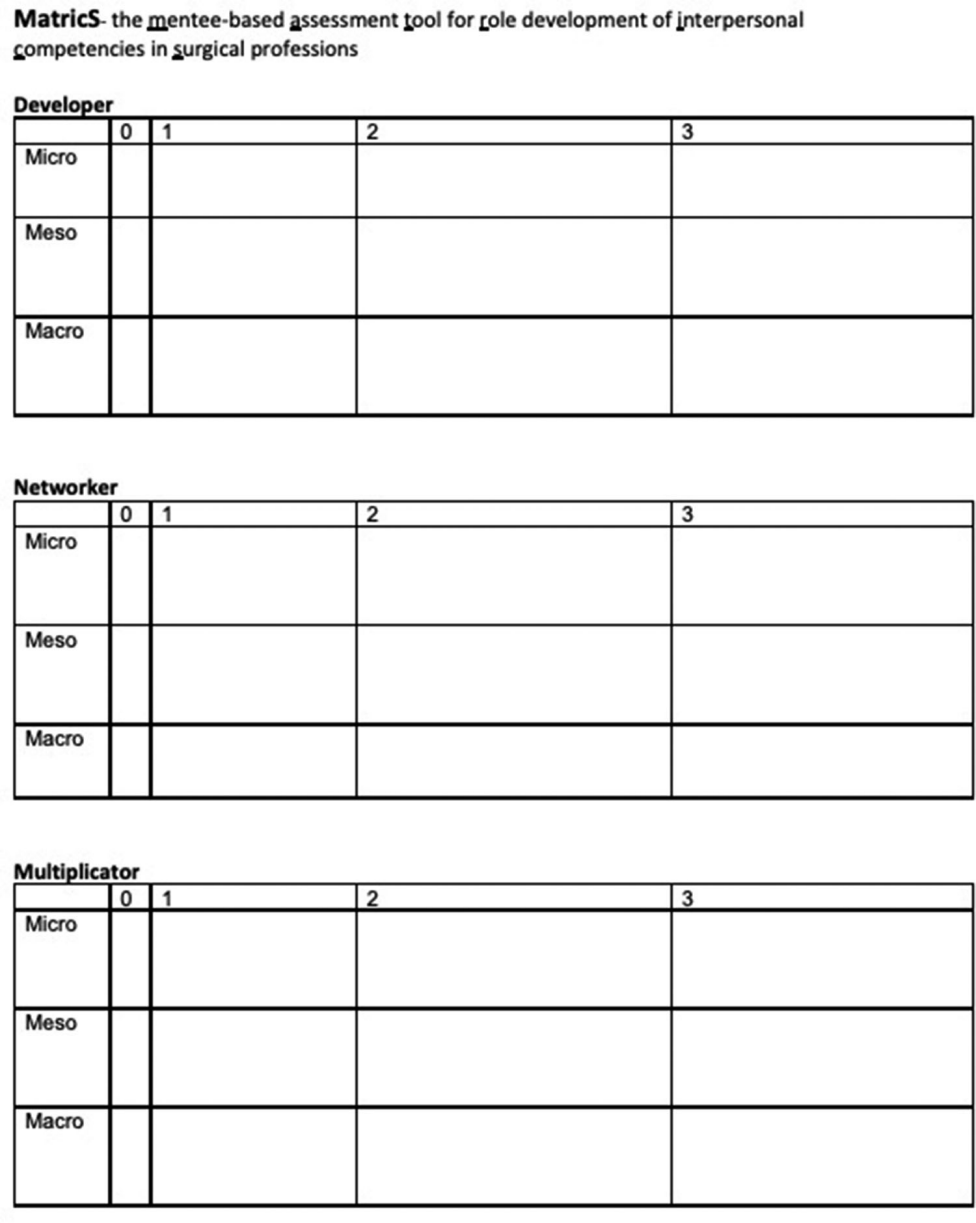
Assessment tool for role development of interpersonal competencies in surgical professions. Using the Learning Objective-based role matrix, mentees and mentors can rate the mentee's competencies on a scale from 0 to 3: 0 indicates no knowledge; 1 indicated basic skills and abilities; 2 indicates knowledge, skills, and abilities consistent with the core learning objectives of the Urology Roadmap mentoring program; 3 indicated knowledge, skills, and abilities corresponding to the core learning objectives and extended learning objectives of the Urology Roadmap mentoring program. Filling the corresponding cell of the matrix with an example of the individual competency is encouraged, but not mandatory.

The quality evaluation of the program is done by a validated tool (15) and results (which were satisfactory) are published elsewhere (13). MatricS is used to assess the professional role development process and focuses on three defined roles (D, developer; N, networker; M, multiplicator) at three levels (private, employer-related, national/international) with four stages of development (0: No knowledge, 1: Basic skills and abilities, 2: Knowledge, skills, and abilities consistent with the core learning objectives of the Urology Roadmap mentoring program, 3: Knowledge, skills, and abilities corresponding to the core learning objectives and extended learning objectives of the Urology Roadmap mentoring program). The definition of each competency within each role and level are presented to all participants at the entry meeting. The matrix (see Figure 1) is subsequently completed by all participants during the entry and exit meeting. The mentee self-assessments from the entry meeting were used by the mentors to individualize their mentoring activities. For our data analysis, the filled assessment forms were collected at both time points immediately after completion by the participants. The forms were then assigned pseudonyms before data analysis. For correlation with mentee self-assessments, the assessments of the respective mentors were recorded alongside, using a content-identical, validated role matrix (13). Individual missing values (3.2%) were imputed using the group mean whenever at least 50% of values from the individual mentor or mentee were available (19), otherwise the corresponding mentor/mentee pair was excluded from analysis. Note that five mentors were in cohort 1 and five mentors were in cohort 2. Two mentors from cohort 1 and their five mentees had to be excluded from correlation analyses because of missing values from the mentor assessments.

Correlation analyses were performed by calculating Pearson coefficients (20), and pre-post-comparisons were done by paired t-tests; significance was assumed at *p* < 0.05, respectively. For sensitivity analyses, we also calculated Spearman's rank correlation coefficients (21) and intra-class correlation coefficients with one-way random effects, absolute agreement and single rater/measurements (22). Means are given ± standard deviations. For graphical depiction of correlations, dashed lines indicate the 95% confidence interval of the respective correlation coefficients. The protocol of the study was approved by the ethical committee of the Bavarian doctors' association.

## Results

**Figure 2:**
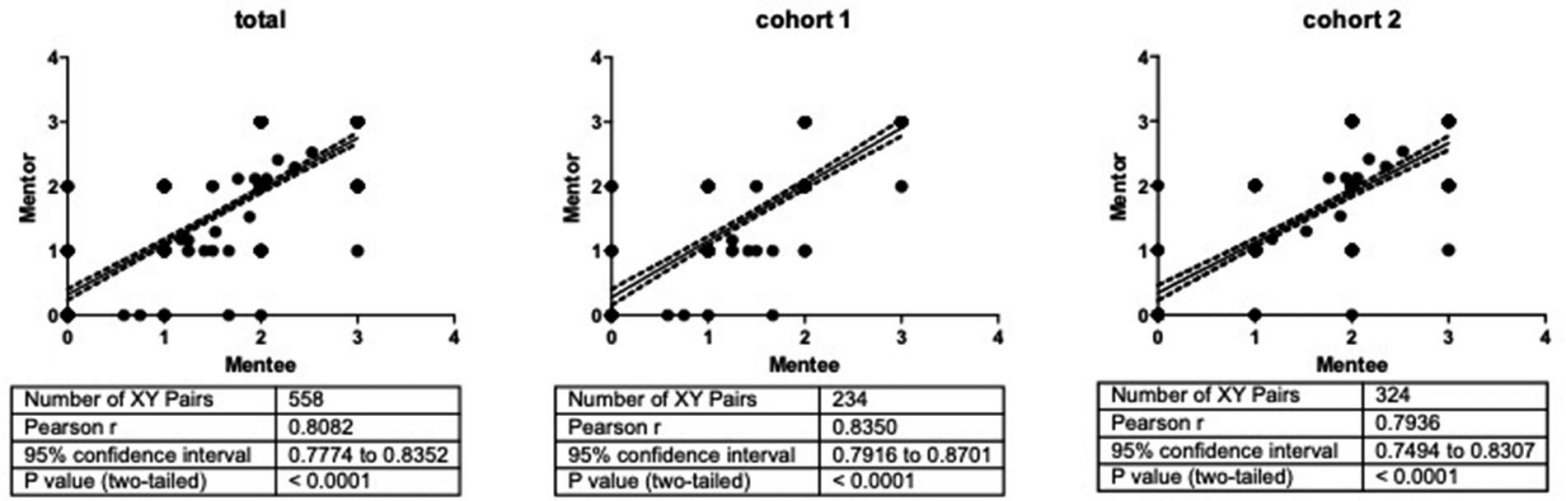
Mentee/mentor assessments correlations. The figure shows Pearson correlations (solid lines) along with their 95% confidence intervals (dashed lines). Individual panels show mentee/mentor correlations for all mentees (left panel) and only mentees of cohort 1 (central panel) or cohort 2 (right panel), respectively. *x*- and *y*-axis units are levels of expertise.

A total of 558 mentee/mentor assessment pairs (from 31 mentees and 10 mentors) could be analyzed. Mentee self-assessments overall correlated well with the objective mentor assessments (Pearson's *r* 0.81, *p* < 0.001; Figure 2, left panel). Significant correlations of the same magnitude were found for both individual cohorts (cohort 1: Pearson's *r* 0.84, cohort 2: Pearson's *r* 0.79, both *p* < 0.001; Figure 2, middle and right panel) as well as for all individual roles (D: Pearson's *r* 0.81, N: Pearson's *r* 0.81, M: Pearson's *r* 0.81, *p* < 0.001 respectively). The correlations and their respective sizes were robust and did not change when non-parametric tests or intraclass correlation tests were used or missing values were not imputed, but instead removed (data not shown, respectively). For more detailed analyses, we also looked at the correlation coefficients of individual mentors' assessments and the self-assessments of their respective mentees (Table 1). We found significant correlations for all mentors, and we could find strong correlations for some mentors (e.g., *r*  > 0.9). For other mentors, estimates of mentee competencies did not correlate so highly with the mentee self-assessments. When we looked at mentor/mentee correlation coefficients from the entry and exit evaluation separately, the observed correlation was stronger at the entry assessment (rentry 0.87, *p* < 0.001) than at the exit assessment (rexit 0.68, *p* < 0.001). Furthermore, individual mentor/mentee correlations were significantly stronger at entry compared to exit (mean rentry 0.83 ± 0.14 vs. mean rexit 0.68 ± 0.14, *p*< 0.01).

**Table 1:** Mentee/mentor assessment correlation coefficients for individual mentors.

	Pearson's *r*	95% confidence interval	Significance level
Mentor A	0.65	0.46–0.78	*p* < 0.001
Mentor C	0.89	0.81–0.93	*p* < 0.001
Mentor D	0.84	0.75–0.90	*p* < 0.001
Mentor F	0.95	0.92–0.97	*p* < 0.001
Mentor G	0.90	0.83–0.94	*p* < 0.001
Mentor H	0.73	0.57–0.83	*p* < 0.001
Mentor I	0.73	0.57–0.83	*p* < 0.001
Mentor J	0.66	0.48–0.79	*p* < 0.001
Mentor K	0.99	0.98–1.00	*p* < 0.001
Mentor L	0.91	0.85–0.95	*p* < 0.001

Note that Mentors A thru F were in cohort 1 and Mentors G thru L were in cohort 2. Mentors B and E were excluded because of missing values from their assessments.

To better understand this finding, we next analyzed the absolute competency changes between entry and exit assessment, i.e., the competency gains of mentees during the program. The mentees acquired competencies overall and in all individual roles indicated by significant increases of corresponding MatricS scores both in the mentee self-assessments (meancompetency level entry 1.22 ± 0.70 vs. meancompetency level exit 1.88 ± 0.81, *p* < 0.001, cohen's *d* 0.87) as well as in the mentor assessments (meancompetency level entry 1.17 ± 0.67 vs. meancompetency level exit 1.98 ± 0.75, *p* < 0.001, cohen's *d* 1.14). All competency gains are shown in Table 2. In general, a pattern could be observed, that at program entry mentee self-assessments showed higher competency levels compared to mentor assessments, while this pattern was reversed at the exit assessments (both differences reaching a significance level *p* < 0.05).

**Table 2:** Mentee competency changes during the mentoring program.

Role	Entry	SD	Exit	SD	Gain	Cohen's d	significance level
**Mentee self - assessment**							
Developer	1.30	±0.64	2.13	±0.74	0.84	1.05	*p* < 0.001
Networker	1.23	±0.70	1.82	±0.81	0.59	0.80	*p* < 0.001
Multiplicator	1.13	±0.77	1.68	±0.83	0.55	0.80	*p* < 0.001
Overall	1.22	±0.68	1.88	±0.79	0.66	0.87	*p* < 0.001
**Mentor assessment**							
Developer	1.26	±0.61	2.14	±0.70	0.89	1.13	*p* < 0.001
Networker	1.16	±0.67	1.93	±0.75	0.77	1.12	*p* < 0.001
Multiplicator	1.08	±0.76	1.88	±0.81	0.80	1.22	*p* < 0.001
Overall	1.17	±0.63	1.98	±0.74	0.82	1.14	*p* < 0.001

Note that differences at entry and exit assessments between mentor and mentee assessments are statistically significant at the level of *p* < 0.05, respectively.

The largest competency gains according to mentee self-assessments as well as mentor assessments were found in the role D. According to the mentor assessments, the majority of mentees (53%) achieved the prespecified target competency per level in >75% of all roles and levels, whereas this was true for only 44% of mentees according to their self-assessments.

## Discussion

We had hypothesized that the use of the novel instrument, MatricS can adequately monitor the professional role development process of residents during an established mentoring program. Our data show that the mentee self-assessments correlate significantly with the respective mentor assessments. As the mentor assessments can be considered the gold standard, correlation coefficients can be interpreted as an indicator of reliability and indicate good reliability given their size of 0.8 (23). Interestingly, we have found a range of correlations varying between individual mentors. This could be for example due to severity/leniency biases (24) of individual mentors as well as over-/underestimation of mentees in their self-assessments. As severity/leniency biases are discouraging over-average performance of the rated individual (25), MatricS in conjunction with the Mentor assessment could be used when new mentors participate in a mentoring program as a basis for their evaluation. Program directors can then direct constructive feedback to their mentors.

We had further investigated, if there was empirical evidence for a Dunning–Kruger effect of mentee self-assessments (26). In line with this, we have indeed found higher mentee self-assessments (compared with the corresponding mentor assessments) at the entry assessment. Along with this finding and a possible transition from over- to underestimation in self-assessments with increasing competencies (27), the mentee self-assessments were significantly lower than the mentor ratings at the exit assessments. However, both mentee and mentor assessments significantly increase over the course of the program with large effect sizes. We conclude therefore, that self-assessment biases are negligible, and the professional role development of the mentees can be objectified by the use of MatricS. This externally valid perspective gives all persons involved in the program the opportunity to recognize which competence increases are still possible in the future. As already described by North et al., the individual competencies of employees can be visualized by competency matrices (28).

Visualizing the three roles of developer, networker and multiplier and the three levels private, employer-related, (inter)national with MatricS can thus help the mentees to get a differentiated picture of their competencies and their increase. Reflection within this framework helps the mentees to better assess themselves, but also contributes to the fact that mentors and the coordination group can provide active support if there are clear deficits in the cohorts. In addition, group mentoring gives mentees the opportunity to compare the role matrices with each other and to motivate each other to expand their competencies. These individual examples of the respective group members named in the role matrix can be seen as instructions on how to achieve an increase in competence in the said role. These synergistic processes are expressly desired and are to be specifically promoted by the coordination circle and mentors (29, 30).

Taken together, our study shows that a new tool MatricS is suitable as an instrument for self-assessment and for making competencies visible.

The present study of course has several limitations that should not go unmentioned. The first limitation is that only three roles have been defined within the role matrix. Other skills and competencies that are important for a physician are not represented in the current role matrix. The three roles of developer, networker and multiplier are the most important roles that represent indispensable competencies in the careers of young urology residents. They should form the basic framework for further studies and be successively supplemented by other roles such as “Leader” or “Professional” of the CanMEDS framework. In future studies it will have to be examined whether an extension by one or more roles additionally enriches the classification for the participants or rather makes it more difficult.

The second limitation is that no independent comparison group was examined within the study. A Rosenthal effect (defined as the influence of the mentor's expectations, beliefs, or biases on the outcome of the program) or a Hawthorne effect (defined as the influence that mere participation in the program has both mentors and mentees) or a multi-factorial natural development cannot be excluded without the investigation of a corresponding control group (especially given the large effect sizes found in this study). Third, the number of included mentors and mentees was limited. The whole program of the “Urology Roadmap” is a big endeavour and only a limited number of people can be included each year. Furthermore, the mentors do the mentoring pro bono and are not compensated for their time, so inclusion is always limited. To get the most wholistic view of the use of the MatricS instrument we therefore tried not to exclude participants when data was missing but rather include the cohort mean to include as many data points as possible. This is a limitation and in further studies we hope to include a version of the MatricS in digital format so that missing values are not an issue.

Fourth, until today few objective evaluations of results such as career outcomes of mentees have been established in mentoring (31). Unfortunately, also our study is not able to provide these outcome data. Because of a potential selection bias (towards intrinsically highly motivated applicants), it would be challenging to assess our program alumni' careers due to the selection of an adequate control group. We believe it is noteworthy though, that after it's inception more than 15 years ago, the program now hosts former mentee/program alumni as active mentors today. However, objective outcome data are useful for monitoring the effectiveness of measures during mentoring program on the basis of defined parameters. It also helps to check whether the goals can be achieved, whether there are structural problems so that an intervention can be made and overall costs could be justified towards the payers.

Nevertheless, our study shows that the professional role development process can be reliably monitored by using MatricS. MatricS scores highly correlate between mentees and mentors, indicating that mentee self-assessments are suitable and sufficient for monitoring. MatricS as an evaluation tool is designed for the medical profession and can thus also be transferred to other specialties. It fills the gap of process evaluation during an ongoing mentoring program. We believe, MatricS helps “mentees at risk” to better achieve their competency goals in the future and hope that future research explores the effectiveness of MatricS-based interventions in mentoring programs. Most important, our findings help to lessen the work burden on senior surgeons and thus can help to increase the acceptance of mentoring programs in surgical disciplines.

## Data Availability

The raw data supporting the conclusions of this article will be made available by the authors, without undue reservation.
